# Proficiency Testing of Virus Diagnostics Based on Bioinformatics Analysis of Simulated *In Silico* High-Throughput Sequencing Data Sets

**DOI:** 10.1128/JCM.00466-19

**Published:** 2019-07-26

**Authors:** Annika Brinkmann, Andreas Andrusch, Ariane Belka, Claudia Wylezich, Dirk Höper, Anne Pohlmann, Thomas Nordahl Petersen, Pierrick Lucas, Yannick Blanchard, Anna Papa, Angeliki Melidou, Bas B. Oude Munnink, Jelle Matthijnssens, Ward Deboutte, Richard J. Ellis, Florian Hansmann, Wolfgang Baumgärtner, Erhard van der Vries, Albert Osterhaus, Cesare Camma, Iolanda Mangone, Alessio Lorusso, Maurilia Marcacci, Alexandra Nunes, Miguel Pinto, Vítor Borges, Annelies Kroneman, Dennis Schmitz, Victor Max Corman, Christian Drosten, Terry C. Jones, Rene S. Hendriksen, Frank M. Aarestrup, Marion Koopmans, Martin Beer, Andreas Nitsche

**Affiliations:** a Robert Koch Institute, Centre for Biological Threats and Special Pathogens 1, Berlin, Germany; b Friedrich-Loeffler-Institut, Institute of Diagnostic Virology, Greifswald–Insel Riems, Germany; c Technical University of Denmark, National Food Institute, WHO Collaborating Center for Antimicrobial Resistance in Foodborne Pathogens and Genomics and European Union Reference Laboratory for Antimicrobial Resistance, Kongens Lyngby, Denmark; d French Agency for Food, Environmental and Occupational Health and Safety, Laboratory of Ploufragan, Unit of Viral Genetics and Biosafety, Ploufragan, France; e Microbiology Department, Aristotle University of Thessaloniki, School of Medicine, Thessaloniki, Greece; f Department of Viroscience, Erasmus Medical Centre, Rotterdam, The Netherlands; g REGA Institute KU Leuven, Leuven, Belgium; h Animal and Plant Health Agency, Addlestone, United Kingdom; i Department of Pathology, University of Veterinary Medicine Hannover, Hannover, Germany; j Department of Infectious Diseases and Immunology, University of Utrecht, Utrecht, The Netherlands; k Artemis One Health Research Institute, Utrecht, The Netherlands; l Istituto Zooprofilattico Sperimentale dell’Abruzzo e Molise G. Caporale, National Reference Center for Whole Genome Sequencing of Microbial Pathogens: Database and Bioinformatic Analysis, Teramo, Italy; m Bioinformatics Unit, Department of Infectious Diseases, National Institute of Health (INSA), Lisbon, Portugal; n National Institute for Public Health and the Environment, Bilthoven, The Netherlands; o Institute of Virology, Charité-Universitätsmedizin Berlin, Berlin, Germany; p Center for Pathogen Evolution, Department of Zoology, University of Cambridge, Cambridge, United Kingdom; Memorial Sloan Kettering Cancer Center

**Keywords:** high-throughput sequencing, external quality assessment, next-generation sequencing, proficiency testing, virus diagnostics

## Abstract

Quality management and independent assessment of high-throughput sequencing-based virus diagnostics have not yet been established as a mandatory approach for ensuring comparable results. The sensitivity and specificity of viral high-throughput sequence data analysis are highly affected by bioinformatics processing using publicly available and custom tools and databases and thus differ widely between individuals and institutions. Here we present the results of the COMPARE [*Co*llaborative *M*anagement *P*latform for Detection and *A*nalyses of (*R*e-)emerging and Foodborne Outbreaks in *E*urope] *in silico* virus proficiency test. An artificial, simulated *in silico* data set of Illumina HiSeq sequences was provided to 13 different European institutes for bioinformatics analysis to identify viral pathogens in high-throughput sequence data. Comparison of the participants’ analyses shows that the use of different tools, programs, and databases for bioinformatics analyses can impact the correct identification of viral sequences from a simple data set. The identification of slightly mutated and highly divergent virus genomes has been shown to be most challenging. Furthermore, the interpretation of the results, together with a fictitious case report, by the participants showed that in addition to the bioinformatics analysis, the virological evaluation of the results can be important in clinical settings. External quality assessment and proficiency testing should become an important part of validating high-throughput sequencing-based virus diagnostics and could improve the harmonization, comparability, and reproducibility of results. There is a need for the establishment of international proficiency testing, like that established for conventional laboratory tests such as PCR, for bioinformatics pipelines and the interpretation of such results.

## Abstract (precis)

Quality management and independent assessment of high-throughput sequencing-based virus diagnostics have not yet been established as a mandatory approach for ensuring comparable results. The sensitivity and specificity of viral high-throughput sequence data analysis are highly affected by bioinformatics processing using publicly available and custom tools and databases and thus differ widely between individuals and institutions.

## INTRODUCTION

High-throughput sequencing (HTS) has become increasingly important for virus diagnostics in human and veterinary clinical settings and for disease outbreak investigations (1–3). Since the introduction of the first HTS platform only about 1 decade ago, sequencing quality and output have been increasing exponentially, and costs per base have decreased. Thus, HTS has become a standard method for molecular diagnostics in many virological laboratories. The relatively unbiased approach of HTS not only enables the screening of clinical samples for common and expected viruses but also allows an open view without preconceptions about which virus might be present. This approach has led to the discovery of novel viruses in clinical samples, such as Bas-Congo virus, associated with hemorrhagic fever outbreaks in Central Africa (2); Lujo arenavirus in southern Africa (3); and a bornavirus-like virus, the causative agent of several cases of encephalitis with fatal outcomes in Germany (4). Considering the potential of HTS to complement or even replace existing “gold-standard” diagnostic approaches such as PCR and quantitative PCR (qPCR), quality assessment (QA) and accreditation processes need to be established to ensure the quality, harmonization, comparability, and reproducibility of diagnostic results. While the computational analysis of the immense amount of data produced requires dedicated computational infrastructure, as well as bioinformatics knowledge or software developed by (bio)informaticians, the interpretation of the results also requires evaluation by an experienced virologist or physician. In many cases, true-positive results may be difficult to discern among large numbers of false-positive results or may be entirely missing from result sets due to false-negative results. Interpretation of results also requires knowledge of anomalies that may arise through sequencing artifacts or contamination.

Proficiency testing (PT) is an external quality assessment (EQA) tool for evaluating and verifying sequencing quality and reliability in HTS analyses. The pioneer in EQA and PT for infectious disease applications of HTS has been the Global Microbial Identifier (GMI) initiative, which has been organizing annual PTs since 2015, focusing on sequencing quality parameters, including the detection of antimicrobial resistance genes, multilocus sequence typing, and phylogenetic analysis of defined bacterial strains (https://www.globalmicrobialidentifier.org/workgroups/about-the-gmi-proficiency-tests) (5). Subsequently, the concept was similarly established regionally for U.S. FDA field laboratories (6, 7).

COMPARE (*Co*llaborative *M*anagement *P*latform for Detection and *A*nalyses of (*R*e-)emerging and Foodborne Outbreaks in *E*urope (http://www.compare-europe.eu/) is a European Union-funded program with the vision of improving the identification of (novel) emerging diseases through HTS technologies. Participating institutions have hands-on experience in viral outbreak investigation. One of the ambitious goals is to establish and enhance quality management and quality assurance in HTS, including external assessment and interlaboratory comparison.

In this study, we present the results of the first global PT offered by the COMPARE network to assess bioinformatics analyses of simulated *in silico* clinical HTS virus data. The viral sequence data set was accompanied by a fictitious case report providing a realistic scenario to support the identification of the simulated virus included in the data set.

## 

### Tools and programs for bioinformatics analysis.

In recent years, numerous tools, programs, and ready-to-use workflows have been established, making metagenomics sequence analyses accessible to scientists from all research fields. Workflows for the typical analysis of HTS data and for the identification of viral sequences are based on the same general tasks and tools, including quality trimming, background/host subtraction, *de novo* assembly, and sequence alignment and annotation. Sequence processing usually starts with obligatory quality assessment and trimming, using programs such as FastQC or Trimmomatic, including the removal of technical and low-complexity sequences or the filtering of poor-quality reads (8, 9). Following these initial steps, many workflows include the subtraction of background reads, e.g., host and bacteria, to reduce the total amount of data and increase specificity, using tools such as BWA (Burrows-Wheeler Alignment Tool) or Bowtie 2 (10, 11). *De novo* assembly of HTS reads into longer, contiguous sequences (contigs), followed by reference-based identification, has been shown to improve the sensitivity of pathogen identification. Such analyses depend heavily on the use of assemblers, such as SPAdes or VELVET, which make use of specific assembly algorithms, such as overlap-layout-consensus graph or de Bruijn graph algorithms (12, 13). Alignment tools such as BLAST, DIAMOND (double-index alignment of next-generation sequencing [NGS] data), Kraken, and USEARCH are among the most important components in bioinformatics workflows for pathogen identification and taxonomic assignment of viral sequences (14–17). Since command-line tools for HTS require specific knowledge in bioinformatics, complete workflows and pipeline approaches have been developed, including ready-to-use Web-based tools, such as RIEMS (*r*eliable *i*nformation *e*xtraction from *m*etagenomic *s*equence data sets), PAIPline (*P*AIPline for the *a*utomatic *i*dentification of *p*athogens), Genome Detective, and others (18–20). Since the COMPARE *in silico* PT focuses on comparing different tools and software programs for bioinformatics analyses, an overview of frequently used programs is given in Table 1. A more extensive overview of virus metagenomics classification tools and pipelines published between 2010 and 2017 can be found at https://compare.cbs.dtu.dk/inventory#pipeline.

**TABLE 1:** Tools and programs for analysis of HTS data used in the COMPARE virus proficiency test^*a*^

Program (reference)	Application	Description/relevance for viral HTS	URL
BWA (10)	Alignment (nucleotide)	Burrows-Wheeler Alignment Tool for efficient alignment of short sequencing reads against a large reference genome. Based on string matching with Burrows-Wheeler transform.	http://bio-bwa.sourceforge.net/
DIAMOND (14)	Alignment (protein)	Double-index alignment of NGS data. Shown to be as much as 20,000 times faster than comparable programs, with high sensitivity.	http://ab.inf.uni-tuebingen.de/software/diamond/
FastQC (9)	Quality control, trimming	Generates base quality scores and sequence contents, sequence length distributions, identification of duplicate or overrepresented sequences, adapter, and k-mer contents.	https://www.bioinformatics.babraham.ac.uk/projects/fastqc/
Kmerfinder (40)	Taxonomic assignment	Online user interface also allows the prediction of human and vertebrate viruses.	https://cge.cbs.dtu.dk//services/KmerFinder/
Kraken (15)	Alignment (nucleotide)	Uses only exact alignments for its taxonomic classification with high speed.	https://ccb.jhu.edu/software/kraken/
MetaPhlAn	Taxonomic assignment	Metagenomic Phylogenetic Analysis is a tool for the taxonomic assignment of microbial communities. High accuracy and speed are supported by only high-confidence matches. Such approaches allow the assignment of 25,000 microbial reads per second but might fail with viral genomes, which often lack common markers and genes.	https://bitbucket.org/biobakery/metaphlan2
MGMapper (41)	Pipeline	Online tool for processing, assigning, and analyzing HTS sequences.	https://bitbucket.org/genomicepidemiology/mgmapper
MIRA	*De novo* assembly	Mimicking Intelligent Read Assembly, an overlap-layout-consensus graph (OLC) assembler for metagenomics data from several sequencing platforms. Assembles the most as well as the largest contigs among *de novo* assembly programs, as well as producing the highest number of contigs that could be assigned to a viral taxon.	https://sourceforge.net/projects/mira-assembler/
NCBI BLAST (16)	Alignment (nucleotide and protein)	Basic local alignment search tool. Offers very sensitive online and stand-alone alignments of nucleotides, translated nucleotides, and protein sequences.	https://blast.ncbi.nlm.nih.gov/Blast.cgi
One Codex (42)	Taxonomic assignment	Web-based data platform for k-mer-based taxonomic classification. Very high degrees of sensitivity and specificity, even when analyzing highly divergent and mutated sequences.	https://www.onecodex.com/
PAIPline (20)	Pipeline	Pipeline for metagenomic analysis of HTS data.	https://gitlab.com/rki_bioinformatics/paipline
QUASR (43)	Pipeline	Combination of several R packages and external software for HTS read analysis. Part of the Bioconductor project.	http://www.bioconductor.org/packages/release/bioc/html/QuasR.html
RIEMS (18)	Pipeline	Pipeline for metagenomics sequence analysis, combining several established programs and tools for pathogen detection in one automated workflow. Separated into a workflow of accurate and fast “basic analysis” and a more sensitive “further analysis.”	https://www.fli.de/en/institutes/institute-of-diagnostic-virology-ivd/laboratories-working-groups/laboratory-for-ngs-and-microarray-diagnostics/
Skewer (44)	Quality control, trimming	Trimming of primer and adapter sequences focusing on the characteristics of paired-end and mate-pair reads. A statistical scheme based on quality values allows the accurate trimming of adapters with mismatches.	https://sourceforge.net/projects/skewer/
SNAP (45)	Alignment (nucleotide)	As much as 10 to 100 times faster than similar alignment programs but offers greater sensitivity due to richer error acceptance.	http://snap.cs.berkeley.edu/
SPAdes, MetaSPAdes (12)	*De novo* assembly	De Bruijn graph assembler. MetaSPAdes specifically addresses the challenges that arise with complex metagenomics data.	http://cab.spbu.ru/software/spades/
Taxonomer (46)	Taxonomic assignment	Web-based tool for nucleotide- and protein-based read assignment. User-friendly interactive result visualization. Based on exact k-mer matching with low error tolerance. Speed as high as ∼32 million reads/min. Furthermore, protein-based read identification offers the detection of divergent viral sequences but is based on exact k-mer matching without error allowance.	https://www.taxonomer.com/
Trimmomatic (8)	Quality control, trimming	Paired-end sequence reads can be cut from technical sequences as adapters, primers, or low-quality bases. Has been shown to improve downstream analyses considerably, for example, *de novo* assembly (increasing contig size up to 77%) and alignment (increasing alignment rates from 7% to 78%).	www.usadellab.org/cms/index.php?page=trimmomatic
USEARCH (17)	Alignment (protein)	Exceptionally high speed for protein or translated nucleotide read alignment. The sensitivity of USEARCH is comparable to that of the NCBI protein BLAST, but USEARCH is ∼350 times faster.	https://www.drive5.com/usearch/
Velvet (13)	*De novo* assembly	Can be used for *de novo* assemblies of short HTS reads using the de Bruijn algorithm. *De novo* assembly using Velvet can be achieved in as little as 14 min.	https://www.ebi.ac.uk/~zerbino/velvet/

a Listed in alphabetical order.

## MATERIALS AND METHODS

### Organization.

The virus PT was initiated by the COMPARE network and organized by the Robert Koch Institute. Participation was free of charge for research groups experienced in analyzing HTS data sets, and the opportunity was announced through email and the COMPARE website.

Participants were asked to analyze an *in silico* HTS data set; the main goal was to identify the viral reads with their bioinformatics tools and workflows of choice and to interpret the results obtained, including final diagnostic conclusions.

An artificial, simulated *in silico* data set of >6 million single-end 150-bp Illumina HiSeq sequences derived from viral genomes, human chromosomes, and bacterial DNA was provided to 13 different European institutes for bioinformatics analysis toward the identification of viral pathogens in high-throughput sequence data. In order to assess how different levels of experience and/or bioinformatics methodologies affect the outputs and interpretation, participants were allowed to use their bioinformatics tools and workflows of choice. Participants were invited to report the PT results via an online survey within 8 weeks (from 16 September 2016 until 16 November 2016). Overall results were anonymized by the organizers, but each participant was provided with the identifier for its own results.

### *In silico* HTS data set.

The simulated *in silico* data set consisted of a total of 6,339,908 reads (Table 2), based on a single-end 150-bp Illumina HiSeq 2500 system run with an empirical read quality score distribution of Illumina-specific base substitutions. The artificial data set was simulated with the ART program (21). Sequences were generated from the Human Genome Reference Consortium Build 38 (GRCh38; NCBI accession numbers CM000663 to CM000686), Acinetobacter johnsonii (NCBI accession number NZ_CP010350.1), Propionibacterium acnes (NCBI accession number NZ_CP012647.1), and Staphylococcus epidermidis (NCBI accession number NZ_CP009046.1). In addition to human and bacterial reads, simulated sequences of four viruses, Torque teno virus (TTV; NCBI accession number NC_015783.1), human herpesvirus 1 (also called herpes simplex virus 1 [HSV-1]; NCBI accession number NC_001806.2), measles virus (MeV; NCBI accession number NC_001498.1), and a novel avian bornavirus (nABV; NCBI accession number JN014950.1) were included in different numbers and with different levels of similarity to known viruses present in databases (Table 2). TTV and HSV-1 were included in the panel as the easiest sequences to identify (with 1,917 and 2,000 reads, respectively, and 100% nucleotide identity with the reference sequences), followed by a slightly altered MeV (1,000 reads, with 82% nucleotide identity to the reference genome) and, as the likely most difficult taxon, nABV (only 500 reads and 55% nucleotide identity to reference sequence JN014950.1).

**TABLE 2:** Composition of the simulated sequence data set^*a*^

Organism	No. of reads	Nucleotide sequence identity with reference (%)
Human	4,834,491	100
Acinetobacter johnsonii	500,000	100
Propionibacterium acnes	500,000	100
Staphylococcus epidermidis	500,000	100
Torque teno virus	1,917	100
Human herpesvirus 1	2,000	100
Measles virus	1,000	82
(Novel) avian bornavirus	500	55

a The total number of reads is 6,339,908.

### Participants.

Thirteen participants applied for the COMPARE virus PT and completed the survey within the given time frame. Participants were registered from Belgium (*n* = 1), Denmark (*n* = 1), France (*n* = 1), Germany (*n* = 4), Greece (*n* = 1), Italy (*n* = 1), The Netherlands (*n* = 2), Portugal (*n* = 1), and the United Kingdom (*n* = 1). The 13 participants represented 13 different institutes or organizations. Information about the participants’ backgrounds is given below (see Table 4).

### Case report.

To simulate clinical relevance and to set the background for evaluation of the bioinformatics results, the following fictitious case report was provided with the data set:

Recently, a 14-year-old boy from Berlin, Germany, was hospitalized with sudden blindness, reduced consciousness and movement disorders. The patient’s mother reported developmental disorders starting 1 year ago, with concentration problems, uncontrolled fits of rage, overall decreasing performance in school and occasional compulsive head nods. Unfortunately, the patient had received neither medical examination nor treatment, but had attended psychological treatment, assuming behavioral problems.

Magnetic resonance tomography of the patient’s brain showed white and gray matter lesions and gliosis. Soon after hospitalization, the patient showed a persistent vegetative state and died.

A sample of the boy’s brain tissue was sequenced using the Illumina HiSeq 2500 platform, resulting in approximately 6 million single end reads of 150 bp each.

This case of subacute sclerosing panencephalitis (SSPE) can be caused by a persistent infection with a mutated MeV (22). However, the symptoms described could also be caused by HSV-1 or bornavirus-like viruses (4, 23).

### Reported PT results.

Results were collected using the Robert Koch Institute’s online survey software VOXCO. The survey contained 23 questions, including general participant information and specifications about the programs used, parameter settings, and computer specifications, as well as the final results of the PT, including an evaluation of the case (see Table S1 in the supplemental material). The responses were collected as single or multiple options from a multiple-choice questionnaire with additional free text for remarks and comments.

### Analysis of PT results.

The results were evaluated based on sensitivity (true-positive rate, i.e., the fraction of true virus reads that were identified), specificity, and the total time of the bioinformatics analysis (Table 3). The time of analysis was evaluated based on the computational time only, without including the time for preparation and discussion of the bioinformatics results. Correlation of the time of analysis with computer and server specifications was based only on the use of online analysis, a personal computer, a server, and a high-performance virtual machine. Although pathogen identification by HTS-related metagenomics should naturally involve experienced qualified health professionals, participants were challenged to attempt an interpretation regardless of the background of the team performing bioinformatics. Given this context, no qualitative or quantitative scoring was performed in this part.

**TABLE 3:** Sensitivity for identified reads of the COMPARE virus proficiency test

Participant^*a*^	Sensitivity				No false-positive result^*b*^	Time of analysis (h)
	Torque teno virus	Human herpesvirus	Measles virus	Avian bornavirus		
1	**1**	0.99	0.21	0	√	3
2	**1**	1.01	0.46	0	√	15.5
3	0.96	0.96	**1**	**1**	√	60
4	0	0.10	0	0	√	216
5	**1**	0.98	**1**	**1**	√	26
6	**1**	0.84	**1**	**1**	–	12
7	0.94	4.00	1.41	0	√	6
8	**1**	1.04	0.99	0	√	7
9	0.29	0.84	0.49	0	√	5
10	**1**	**1**	**1**	0	√	48
11	**1**	**1**	**1**	0	√	14
12	**1**	**1**	1.02	0.23	√	18
13	1.02	0.90	0.34	0	√	48

a Numbered randomly.

b √, no false-positive result; –, false-positive result(s).

### Availability of data.

The data set used in this study has been uploaded to the European Nucleotide Archive with the study accession number PRJEB32470.

## RESULTS

### PT results.

**FIG 1:**
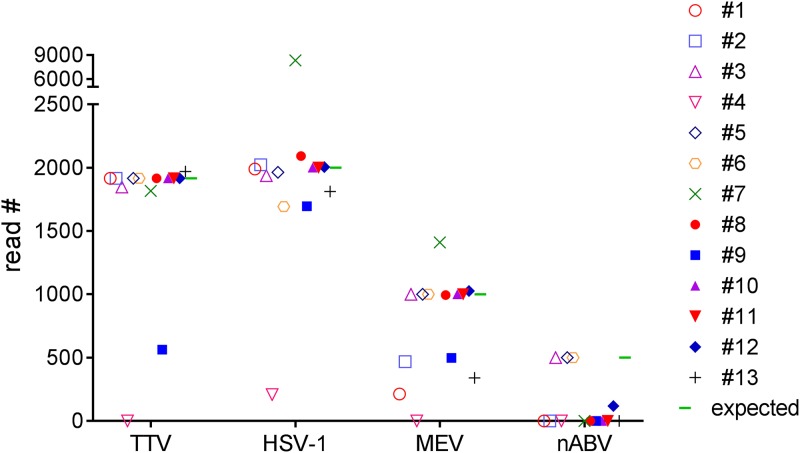
Numbers of Torque teno virus (TTV), human herpesvirus 1 (HSV-1), measles virus (MeV), and novel avian bornavirus (nABV) reads identified by participants 1 to 13.

The results of the PT were evaluated based on sensitivity, specificity, total turnaround time, and interpretation of results (Table 3). HSV-1 was identified by all participants (Tables 3 and 4; Fig. 1). For most of the participants, the identified read numbers for HSV-1 were complete or nearly complete (actual HSV-1 read count, 2,000). One participant identified more reads of HSV-1 than were present in the data set (participant 7; 8,361 reads identified).

**TABLE 4:** Interpretation of bioinformatics results

Participant	Results of:		Participant’s background
	Bioinformatics^*a*^	Diagnostics	
1	TTV, HSV-1, MeV	HSV-1	Bioinformatics
2	TTV, HSV-1, MeV	HSV-1	Food and environmental health
3	TTV, HSV-1, MeV, nABV	SSPE/HSV-1	Veterinarian, virology
4	HSV-1	HSV-1	University, virology
5	TTV, HSV-1, MeV, nABV	nABV	Virology
6	TTV, HSV-1, MeV, nABV	nABV	Medical research
7	TTV, HSV-1, MeV	SSPE	Animal and plant health
8	TTV, HSV-1, MeV	SSPE	Veterinarian, virology
9	TTV, HSV-1, MeV	SSPE	Public health
10	TTV, HSV-1, MeV	SSPE	Public health
11	TTV, HSV-1, MeV	SSPE	Public health and environment
12	TTV, HSV-1, MeV, nABV	SSPE/HSV-1	Diagnostics, virology
13	TTV, HSV-1, MeV	SSPE	Virology

a Abbreviations: TTV, Torque teno virus; HSV-1, human herpesvirus 1; MeV, measles virus; nABV, novel avian bornavirus; SSPE, subacute sclerosing panencephalitis.

TTV (actual read count, 1,917) and MeV were identified by all participants except for one (participant 4) (Tables 3 and 4; Fig. 1). For TTV, the read numbers identified were complete or almost complete for all participants, with the exception of participant 9, who was able to identify only 29% of the TTV reads. For the mutated MeV (actual read count, 1,000), 7 of the 13 participants were able to identify complete or almost complete read numbers (participants 3, 5, 6, 8, 10, 11, and 12), whereas 4 participants (participants 1, 2, 9, and 13) identified only 21%, 46%, 49%, and 34% of the total number of 1,000 reads, respectively (Table 3). Participant 4 was unable to identify MeV, and participant 7 assigned too many reads (1,411) as originating from the mutated MeV.

The divergent nABV (actual read count, 500) proved to be the most challenging target and was identified by only four of the participants (participants 3, 5, 6, and 12) (Tables 3 and 4; Fig. 1). The overall specificity for all bioinformatics workflows was high, with only participant 6 identifying 43 reads as a chordopoxvirus, a false-positive result.

The total times of analysis differed widely, from 3 h (participant 1) to 216 h (15 h of online analysis, with an additional 201 h waiting for server availability; participant 4) (Table 5). Most workflows were calculated on a server system; two participants used a personal computer, and two used a virtual machine. One calculation was executed through an external public server.

**TABLE 5:** Total time of computational analysis, maximum computer/server specifications, and reference databases used^*a*^

Participant	Time of analysis (h)	Database	Operating system	CPU	CPU MHz	RAM (GB)
1	3	NCBI nt	UNIX	VM	VM	VM
2	15.5	NCBI nt	Ubuntu 16.04 LTS	56	1,270	378
3	60	NCBI nt/nr	CentOS 6	24	2,400	64
4	216	NCBI nt	Windows XP	Intel core i5	2,300	8
5	26	NCBI viral db	OS X	2	NA	NA
6	12	NCBI nr	Ubuntu 14.04	32	2,000	503
7	6	ViPR and NCBI nt	BioLinux Ubuntu 14.04	8	3.6	16
8	7	NCBI nt	CentOS 6.5	64	2,300	250
9	5	NCBI nr	Ubuntu 12.04.5	NA	3,800	50
10	48	NCBI nt	CentOS 6.5	2 × AMD Opteron	2,200	32
11	14	NCBI nt/nr	RHEL	VM, variable	VM, variable	VM, variable
12	18	NCBI viral db	Linux Mint	Intel Xenon X5650	6 × 2.67 Ghz	25
13	48	NCBI nt	Ubuntu 14.04.4 LTS	2 × AMD Opteron 6174	24 × 2.2 GHz	128

a nr, nonredundant; nt, nucleotide; db, database; VM, virtual machine; NA, not available.

**FIG 2:**
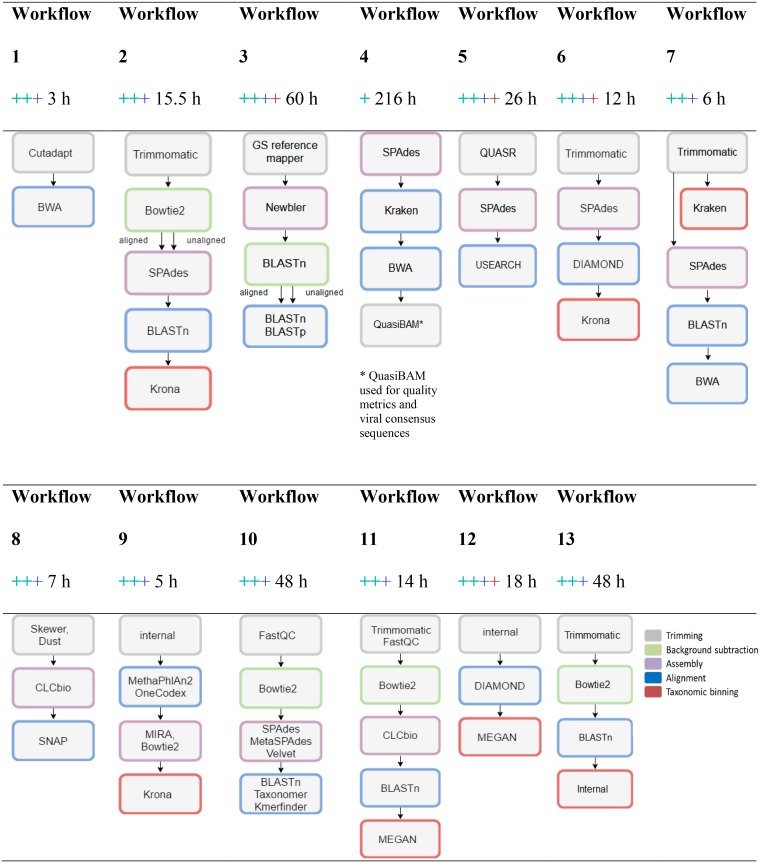
Simplified comparison of different bioinformatics workflows for virus identification used in the COMPARE virus proficiency test. Colored plus signs indicate the identification of human herpesvirus (turquoise), Torque teno virus (turquoise), measles virus (blue), or avian bornavirus (red).

Most of the workflows used in the COMPARE virus PT were quite similar, with the same basic tasks applied in different orders (Fig. 2). Most workflows started with trimming and quality filtering, followed by the subtraction of background reads, the assembly of remaining reads, and a final reference-based viral read assignment (Fig. 1). The databases used were custom-made or full databases from NCBI nt/nr GenBank (participants 1 to 4, 6 to 11, and 13). Participants 5 and 12 used viral sequences from NCBI GenBank only, while participant 7 also included a database for human-pathogenic viruses (ViPR) (https://www.viprbrc.org/brc/home.spg?decorator=vipr).

All groups were also asked to correlate the results based on the bioinformatics analysis with the clinical symptoms described in the case report (Table 4). HSV-1 was suspected as the disease-causing agent by three groups, and MeV was identified by six groups. An MeV infection with HSV-1 possibly affecting the course of disease was named by two groups. nABV was interpreted as the single causative agent by two groups.

## DISCUSSION

HTS-based virus diagnostics requires complex multistep processing, including laboratory preparation, assessment of the quality of sequences produced, computationally challenging analytic validation of sequence reads, and postanalytic interpretation of results. Therefore, not only comprehensive technical skills but also bioinformatic, biological, and medical knowledge is of paramount importance for proper analyses of HTS data for virus diagnostics.

HTS data can comprise several hundred thousand to many millions of reads from a single sequenced sample. Handling and analyzing such amounts of data pose computational challenges and currently require know-how and expertise in bioinformatics. Depending on the laboratory procedure, identification of viral reads from clinical metagenomics data is negatively affected by low virus-to-host sequence ratios and high viral mutation rates, making reference-based sequence assignments for highly divergent viruses challenging (24).

*In silico* bioinformatics analysis of HTS data can be separated into an analytic and a postanalytic step. The analytic step includes the processing of sequence reads with software tools or scripts assembled into workflows and pipelines. The postanalytic step is evaluation of the results obtained from the bioinformatics analysis with regard to pathogen identification, often involving interpretation by an experienced, qualified health professional to correlate bioinformatics results with clinical and epidemiological patient information.

The bioinformatics analysis and the technical identification of viral reads from the HTS data set were shown to have decreasing success as sequences became more divergent from reference strains, as exemplified by MeV, with 82% identity on the nucleotide level to its closest relative, and nABV, with just 52% identity on the nucleotide level to other bornaviruses, which was identified by only 4 of the 13 participants. MeV and TTV were missed by participant 4, whose analysis was based on the Kraken tool and an in-house workflow. Kraken is known to align sequence reads to reference sequences with high specificity and low sensitivity, making the alignment of mutated and divergent virus reads difficult (15). Since Kraken employs a user-specific reference database, TTV may have been absent from the custom database; Kraken was also used by participant 7, which was able to identify both MeV and TTV. It is noted that the use of different databases is an obstacle in bioinformatics analysis of HTS data. To date, there have been unified, curated virus reference databases only for influenza viruses (EpiFlu) (25), HIV (26) and human-pathogenic viruses (ViPR) (27). Recently, viral reference databases for bioinformatics analysis of HTS data have been developed (https://hive.biochemistry.gwu.edu/rvdb, https://rvdb-prot.pasteur.fr/) (28). NCBI offers the most extensive collection of viral genomes, but the lack of curation and verification of submitted sequences often leads to false-positive and false-negative results. To overcome such problems, reference-independent tools for virus detection in HTS data have been developed, making the discovery of novel viruses feasible without any knowledge of the reference genome (29). All of the participants that were able to identify the divergent nABV used workflows based on protein alignment approaches, including BLASTx/p, USEARCH, and DIAMOND, which are known to be highly sensitive (14, 17). The identification of such highly divergent viruses is still challenging and cannot be accomplished by workflows with nucleotide-only reference-based alignment approaches. DIAMOND, which became available in 2015, was specifically designed for such sensitive analysis of HTS data at the protein level and is as much as 20,000 times faster than BLAST programs. Compared to other alignment tools, which seem to have a trade-off between speed and sensitivity, DIAMOND offers superior sensitivity for the detection of mutated and divergent viral sequences (14). However, the detection of such highly divergent viral sequences in patient samples is rare, and virus discovery is not a routine part of clinical virus diagnostics.

In terms of specificity, all workflows were highly specific; only workflow 6 showed the identification of a chordopoxvirus that was not present in the data set. Such false-positive results, as well as the excessive number of HSV-1 and MeV reads found by participant 7 (8,361 of 2,000 reads and 1,411 of 1,000 reads, respectively), can derive, for example, from low-complexity reads in the data set that are aligned to low-complexity or repetitive sequences of the viral reference genomes, from inappropriate matching score limits during filtering, or from inappropriate algorithm parameters. Furthermore, custom databases and viral references from NCBI can include sequences of human origin that can lead to false-positive results, resulting, in some cases, in nonreporting of other matches due to default algorithm reporting limits.

The total times of all workflows differed widely, from only 3 h to 216 h (15 h for the analysis and 201 h waiting for available servers). One of the fastest participants was participant 1, which needed only 3 h to perform the calculations on a scalable high-performance national virtual machine, whereas the slowest workflow (participant 4; 216 h) involved calculation on a personal computer through an external public server where bioinformatics software jobs are queued among many other users (Fig. 1; Table 5). However, participant 5 also performed analysis on a notebook but within a much shorter time (26 h). Overall, workflows exclusively specified for virus detection or using only a viral or RefSeq database did not clearly correlate with shorter times than workflows with full metagenomics analyses. However, the specific composition of each database was not provided. To finally evaluate the performance of each bioinformatics workflow with regard to the time of analysis, all workflows should be run on the same computer system, but such standardization was not practical for this PT evaluation.

The COMPARE virus PT has further shown that both analytic work and postanalytic evaluation are of importance, since similar analytic results can be interpreted very differently, depending on the analyzing participant. Unlike standard routine virus diagnostic approaches such as PCR, where a medical hypothesis of relevance tests either positive or negative, HTS offers an extensive and largely unbiased catalogue of results. The etiological agent of a patient sample can be masked by false-positive results, sequencing contaminants, commensal viruses of the human virome, or viruses of yet unknown importance. Furthermore, the causative viral agent of a disease may be present in very low read numbers, because viral loads may be low, depending on the timing of sampling and the sample matrix. RNA viruses, among which are the most pathogenic human viruses, usually have smaller genomes than DNA viruses (30, 31). Therefore, low read numbers from an RNA virus might be dismissed, resulting in a false-negative result. To assess sequencing results, some workflows and pipelines use cutoffs for read numbers so as to reduce false-positive results, but they may in the process make the detection of low-read-number matches less likely.

Since the analysis of HTS data for virus diagnostics requires bioinformatics as well as virological knowledge, collaboration between the two disciplines has been emphasized (32). Furthermore, automated pipelines for HTS-based virus diagnostics with unbiased evaluation of the pathogenicity and relevance of the pathogen detected have been implemented; these can help harmonize the analysis and interpretation of HTS sequence results (33).

A robust approach to viral diagnostics using HTS requires further refinement and validation. The COMPARE *in silico* PT is limited by the low complexity of the simulated data set. *In vivo* sequence data sets can comprise a highly diverse background and microbiome of the host, further increasing the difficulty of identifying viral reads. Further proficiency schemes with *in vivo* data sets and samples and wider collaboration are required to make progress. A second *in silico* PT organized by the COMPARE network has focused on the interpretation of the significance of foodborne pathogens in a simulated data set (unpublished data). Again, the interpretation of the results was shown to be one of the most diverse and critical points in HTS data analysis. Furthermore, third-generation sequencing technologies, such as MinION from Oxford Nanopore Technologies, are becoming available in many laboratories and field settings due to low cost and short sequencing times (34–36). However, analysis tools developed for second-generation sequencing technologies, such as the Illumina system, may not be applicable for third-generation sequencing data, due to the low sequencing accuracy of approximately 85% and the length of the sequences, which can be as long as 2 Mbp (37–39). Consequently, future PTs should also include the use of third-generation sequencing technologies, since those are likely to become part of routine laboratory diagnostics in the future.

### Conclusion.

The present availability of external quality assessment for HTS-based virus identification is limited. The COMPARE *in silico* virus PT has shown that numerous tools and different workflows are used for virus analysis of HTS data and that the results of such workflows differ in sensitivity and specificity. At present, there are no standard procedures for virome analyses, and the sharing, comparison, and reliable production of the results of such analyses are difficult.

Finally, there is a clear need for creating updated, highly curated, free, publicly available databases for harmonized identification of viruses in virome data sets, as well as mechanisms for conducting continuous ring trials to ensure the quality of virus diagnostics and characterization in clinical diagnostic and public and veterinary health laboratories.

## Supplementary Material

Supplemental file 1
