# Supplementary material for: Proficiency Testing of Virus Diagnostics Based on Bioinformatics Analysis of Simulated In Silico High-Throughput Sequencing Data Sets
Source: J Clin Microbiol. 2019 Jul 26;57(8):e00466-19. doi: 10.1128/JCM.00466-19 (PMC6663916; doi:10.1128/JCM.00466-19)
Supplement: Supplemental file 1 [file JCM.00466-19-s0001.pdf]

Table S1: Questions included in the Survey

Question #	Question content
**1**	Information on the participant (contact name/institute name/country/email/phone number)
**2**	Which platform did you use for your bioinformatics analyses of the sequence data? PC/notebook/server/cloud computing/online-based analyses/external analyses
**3**	Please specify the program or software used for your NGS data analyses: Internally developed software/externally developed software (e.g. SURPI)/online-based analyses (e.g. MG-RAST)/external analyses
**4**	If your bioinformatics analysis was performed on your PC/notebook, please indicate your computer system features: Operating system, CPU, CPU MHz, RAM, hard disc storage
**5**	If your bioinformatics analysis was performed on a server system, please indicate your server system features: Operating system, CPU, CPU MHz, RAM, hard disc storage
**6**	If you used a cloud system for your bioinformatics analysis (e.g. Amazon web services), please indicate the provider of the cloud system: Cloud provider/computer capacity requested/used
**7**	If you uploaded your data for online/external analysis (e.g. MG-RAST.com, virfind.org), please indicate the provider of the analysis: provider name
**8**	Please explain briefly the tasks of your bioinformatics workflow
**9**	Which program did you use for trimming your sequence data? Trimmomatic/Cutadapt/other
**10**	Indicate the tasks used in your trimming process. If possible, comment on the parameters used for these tasks: Quality filtering/adapter/primer removal/length filtering/complexity filtering/duplicate reads removal
**11**	For subtraction of background reads, which databases did you use? None/human/bacterial/artificial/parasites/plant/other
**12**	From where did you obtain your database(s)? NCBI/Uniprot/other
**13**	Did you edit your databases for subtraction of background reads, e.g. choosing reference sequences or building optimized indices?
**14**	For subtraction of background reads, which program/software/tool did you use? If possible, indicate parameters used, e.g. default, local or global alignment, sensitivity, version
**15**	For your assembly, which program did you use? If possible, please indicate parameters, e.g. default, k-mer length, sensitivity, version
**16**	For identification of your viral reads, which program did you use? If possible, please indicate parameters, e.g. default, sensitivity, e-value
**17**	For interpretation of your results (taxonomic binning), which program did you use?
**18**	Before generating your final results with the workflow indicated above, did you try one or more workflows that led to no or unsatisfying results? Please explain briefly the methods and workflows you have tried.
**19**	For your analysis that led to your final results, how much time in total did you need? Days/hours/minutes
**20**	If possible, indicate time needed for different tasks of your workflow. If possible, also indicate number of reads used in each task: Trimming/background subtraction/assembly/virus alignment/BLAST/taxonomic binning/final analysis/other

**21**	Which viral sequences did you find in your data? For each virus, specify the closest related virus species, including nucleotide and protein identities: virus species/nucleotide identity/protein identity/number of reads/number of contigs
**22**	What do you think is the causative agent of the disease explained in the case report and why?
**23**	Anything else you consider important
